# Prognosis and pain dissection of novel signatures in kidney renal clear cell carcinoma based on fatty acid metabolism-related genes

**DOI:** 10.3389/fonc.2022.1094657

**Published:** 2022-12-09

**Authors:** Ruifeng Ding, Huawei Wei, Xin Jiang, Liangtian Wei, Mengqiu Deng, Hongbin Yuan

**Affiliations:** ^1^ Department of Anesthesiology, Changzheng Hospital, Second Affiliated Hospital of Naval Medical University, Shanghai, China; ^2^ Jiangsu Province Key Laboratory of Anesthesiology, Xuzhou Medical University, Xuzhou, China

**Keywords:** kidney renal clear cell carcinoma, fatty acid metabolism, prognostic signature, nomogram, tumor microenvironment, cancer pain

## Abstract

Renal cell carcinoma (RCC) is a malignant tumor that is characterized by the accumulation of intracellular lipid droplets. The prognostic value of fatty acid metabolism-related genes (FMGs) in RCC remains unclear. Alongside this insight, we collected data from three RCC cohorts, namely, The Cancer Genome Atlas (TCGA), E-MTAB-1980, and GSE22541 cohorts, and identified a total of 309 FMGs that could be associated with RCC prognosis. First, we determined the copy number variation and expression levels of these FMGs, and identified 52 overall survival (OS)-related FMGs of the TCGA-KIRC and the E-MTAB-1980 cohort data. Next, 10 of these genes—FASN, ACOT9, MID1IP1, CYP2C9, ABCD1, CPT2, CRAT, TP53INP2, FAAH2, and PTPRG—were identified as pivotal OS-related FMGs based on least absolute shrinkage and selection operator and Cox regression analyses. The expression of some of these genes was confirmed in patients with RCC by immunohistochemical analyses. Kaplan–Meier analysis showed that the identified FMGs were effective in predicting the prognosis of RCC. Moreover, an optimal nomogram was constructed based on FMG-based risk scores and clinical factors, and its robustness was verified by time-dependent receiver operating characteristic analysis, calibration curve analysis, and decision curve analysis. We have also described the biological processes and the tumor immune microenvironment based on FMG-based risk score classification. Given the close association between fatty acid metabolism and cancer-related pain, our 10-FMG signature may also serve as a potential therapeutic target with dual effects on ccRCC prognosis and cancer pain and, therefore, warrants further investigation.

## Introduction

Renal cell carcinoma (RCC) originates in tubular epithelial cells, occupying approximately 2%–3% of adult malignancies (1). For several decades, the incidence and mortality of RCC have been on the rise. According to the International Agency for Research on Cancer, 431,288 new cases of clear-cell RCC (ccRCC) were diagnosed and 179,368 deaths related to this cancer were recorded worldwide in 2020 (2). The majority of deaths associated with kidney cancer are caused by ccRCC, which is the most common subtype (3). The survival rate after treatment for early-stage RCC is 60–70%, while advanced RCC usually has a poor prognosis, of which the 5-year survival is<10% (4). Therefore, it is clinically significant to predict prognosis and provide guidance for personalized treatment by exploring potential markers to improve overall survival of patients.

More and more evidence shows that metabolic changes play an explanatory role in tumor progression (5). Although increased lipid synthesis has received less attention than aerobic glycolysis, it has recently been recognized as another important metabolic abnormality required for carcinogenesis (6). There is growing evidence to suggest that upregulation of several enzymes involved in fatty acid metabolism is a universal metabolic marker in cancer cells (7). In many cancers, lipids are ingested and stored to meet the energy needs of tumor cells, which are supplied with energy by fatty acids through the process of β-oxidation (8). ccRCC is characterized by a high rate of mutation of genes that control metabolism; therefore, this cancer is also thought to be driven by metabolic changes (9). In fact, it is known that ccRCC cells accumulate a large amount of lipids and exhibit abnormal fatty acid metabolism, which is correlated with clinical outcomes (10).

Pain is one of the most common and bothersome symptoms in cancer patients. Across all stages of cancer, 50.7% of patients experience pain; in particular, 66.4% of cancer patients in the advanced stage experience pain (11). Uncontrolled pain can contribute to poor physical and emotional well-being. It is widely accepted that cancer pain is caused by nociceptive, inflammatory, and neuropathic mechanisms (12). It is essential to note that fatty acid metabolism not only has an impact on cancer development but also has an effect on pain development. As shown in the study by Koundouros et al., an increase in the levels of arachidonic acid and eicosanoids can promote cell proliferation (13). Furthermore, the role of arachidonic acid and its metabolite prostaglandin in inflammation and pain has been demonstrated (14). Both anandamide hydrolase and monoacylglycerol lipase are endocannabinoid-degrading enzymes, and inhibitors of these enzymes can reduce pain by blocking the metabolism of anandamide and 2-arachidonic glycerol, while increasing endogenous levels of fatty acid amides. Interestingly, inhibitors of these enzymes, on their own or in combination with other drugs, have shown therapeutic potential in a variety of cancers (15, 16). Thus, further investigation of the role of fatty acid metabolism-related genes (FMGs) in ccRCC might be useful for better prediction of patient prognosis and pain management.

In this study, we constructed a fatty acid-related signature to evaluate the prognosis of RCC. Potential relationships between this signature and the immune microenvironment were investigated. Moreover, we attempted to determine the potential association between these genes and cancer pain, as this could provide new insights into personalized cancer therapy.

## Materials and methods

### Data source

Transcriptome sequencing (mRNA) data, along with detailed clinical information about RCC patients, were acquired from The Cancer Genome Atlas (TCGA) database, the E-MTAB-1980 cohort (17) in the EMBL-EBI database, and the GSE22541 cohort in the Gene Expression Omnibus (GEO) database. Altogether, we obtained data for 535 samples from the TCGA-KIRC database, 101 samples from the E-MTAB-1980 cohort, and 68 samples from the GSE22541 cohort.

### Screening of FMG-associated genes

A predefined set of FMGs was obtained from the Molecular Signature Database (MSigDB, v7.4) (18). We identified three relevant sets of FMGs, namely, KEGG fatty acid metabolism pathway genes, hallmark fatty acid metabolism genes, and reactome fatty acid metabolism genes. After deleting duplicates from these three sets of genes, 309 reliable records were obtained. Furthermore, we performed intersection analysis of these 309 genes with three ccRCC cohorts, and finally obtained 291 genes for follow-up studies (**Supplementary Figure 1**, **Supplementary Table 1**).

### Identification of mutated and differentially expressed genes

The UCSC Xena database (19) was used to obtain the copy number variation (CNV) information of the TCGA-KIRC patients. Then, we calculated and summarized the most significant results of CNV frequencies for these FMGs. Differential expression genes (DEGs) between normal kidney group and KIRC group were analyzed by “limma” package in R, and genes with fold change > 1.50 and *P*< 0.05 were considered to be differentially expressed.

### Construction and validation of risk scores

Univariate Cox regression analysis was used to identify FMGs associated with overall survival (OS) in the TCGA-KIRC and E-MTAB-1980 datasets (*P*< 0.01), and the least absolute shrinkage and selector operation (LASSO) analysis was used to analyze overlapping gene sets with the “glmnet” package in R (20). The prognostic genes were determined by the best penalty parameter λ, and 10 optimal FMGs were screened out. The expression levels between normal kidney group and KIRC group and Kaplan-Meier (K-M) analysis results were also respectively shown base on TCGA-KIRC cohort. Furthermore, the fatty acid metabolic index (FMI) was calculated by adding the expression and corresponding coefficients of the FMGs for each RCC patient. In order to make the results more intuitive, MinMax variation was used to adjust FMI by using the following formula.


$$Adjust\;FMI=\frac{x_{i}-min\left(x_{i}\right)}{max\left(x_{i}\right)-min\left(x_{i}\right)}$$

The median cut-off value of FMI was used to classify patients, and prognostic performance was evaluated by K-M analysis and time-dependent receiver operating characteristic (ROC) analysis.

### Comprehensive assessment of FMI in patients

The association of FMG-based risk scores with clinical features was analyzed based on adjusted FMI values to assess the clinical usability of FMGs. The factors included age, T/N/M stage, and tumor grade.

### Construction and evaluation of an FMG-based clinicopathologic nomogram

Univariate and multivariate Cox regression analyses were performed to explore the prognostic value of FMI. A nomogram combining the clinical features of RCC and FMG-based risk score was developed. To evaluate the performance of nomogram, calibration curve, ROC curve and decision curve analysis (DCA) were performed.

### Functional enrichment analysis of the FMI groups

To further characterize the biological processes in different FMI groups, gene set enrichment analysis (GSEA) was performed. Enrichment results with *P*< 0.05 as well as FDR< 0.1 were considered statistically significant.

### Evaluation of the immunogenomic landscape of RCC

Immune checkpoints are new target molecules in immunotherapy for RCC. In this study, the immune checkpoints were compared between the FMI groups in the three cohorts to evaluate the potential application of these immune checkpoints for FMI-based immunotherapy. The candidate checkpoints identified were PDCD1, IL2RA, MICB, SELP, CX3CL1 and EDNRB.

Since the tissue samples used in transcriptome sequencing are not composed of single cells, the heterogeneity of these samples is inevitable. Therefore, the gene expression profile data may also reflect changes in the cell components in the tissue. In this study, xCell tool was used to predict the immune microenvironment typing of gene expression profile data, and further compared the expression differences of cell subsets between different groups.

### Analysis of sensitivity to chemotherapy

Based on the Genomics of Drug Sensitivity in Cancer (GDSC) database, we performed the “pRRophetic” package in R to predict semi-inhibitory concentrations (IC50) of ccRCC chemotherapeutic drugs between different groups.

### Validation of genes included in the risk model

Immunohistochemical (IHC) staining was performed with antibodies against FASN (D162701, BBI), ACOT9 (D121491, BBI), FAAH2 (D122328, BBI), and PTPRG (GB114422, Servicebio) to validate the expression of risk model-related genes in 10 paired tumor and normal tissues from the Naval Medical University cohort. The procedure for IHC was based on a previous protocol (21). Three independent blind observers analyzed the images by using ImageJ Software (ImageJ, Marlyand, USA), and sum of area and integrated option density (IOD) were measured. The mean integrated option density was calculated by dividing the IOD sum by the area sum.

### Statistical analysis

Unless otherwise stated, statistical significance was considered significant at *P*< 0.05 and two-sided tests.

## Results

### Construction of the FMG-related signature for ccRCC

**Figure 1:**
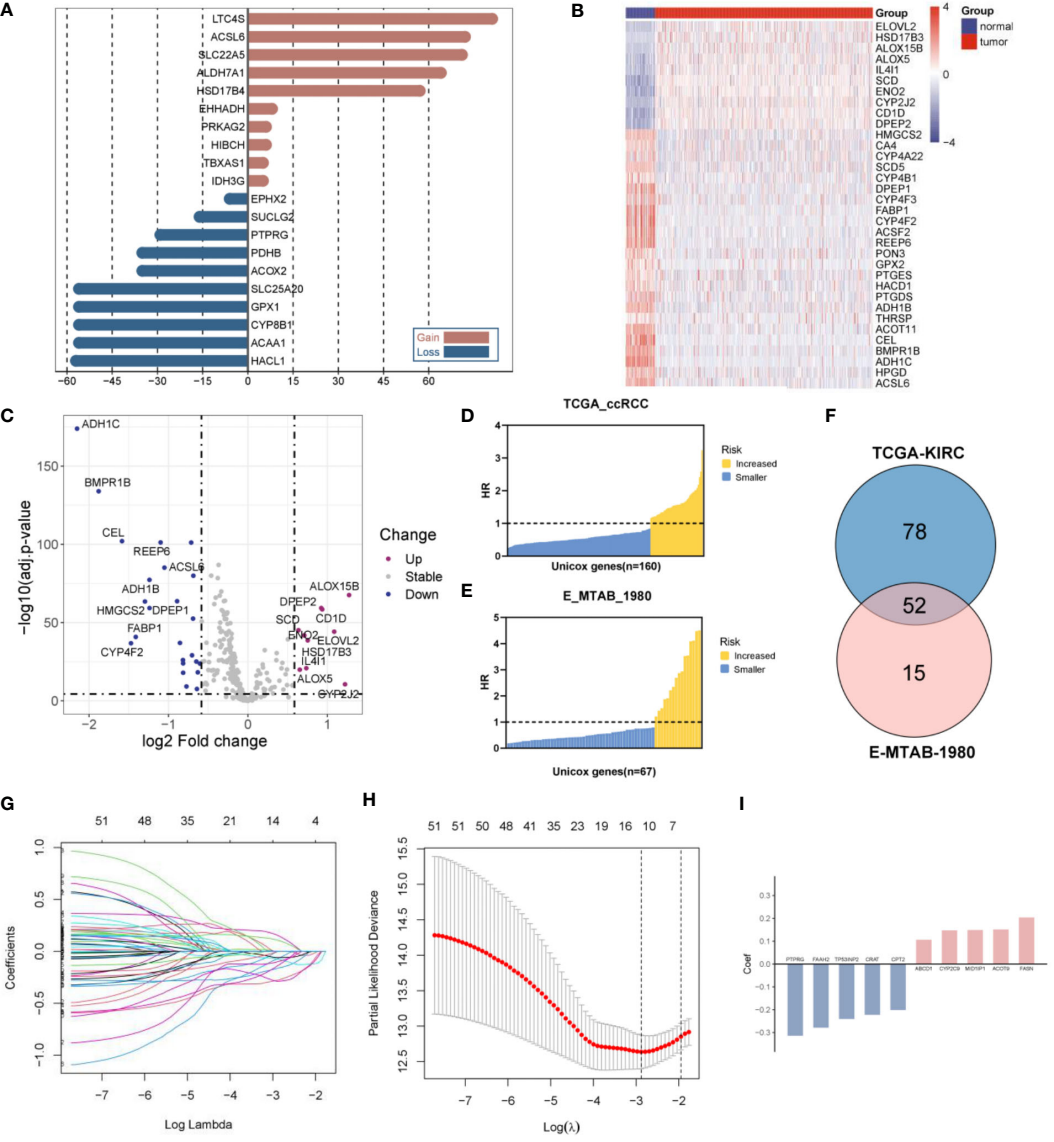
Construction of a fatty acid metabolism-related signature in ccRCC patients. **(A)** The frequency of the top 10 genes with amplified or deleted CNVs of FMGs in the TCGA-KIRC cohort. **(B)** Heatmap analysis of 34 DEGs among FMGs. **(C)** Volcano plot depicting the distribution of DEGs. **(D)** 160 prognostic FMGs in the TCGA-KIRC dataset. **(E)** 67 prognostic FMGs in the MTAB dataset. **(F)** Venn plot identifying 52 overlapping prognostic FMGs. **(G)** LASSO Cox regression analysis of the 52 prognostic FMGs. **(H)** Plot depicting partial likelihood deviance of the LASSO regression. **(I)** Corresponding coefficients of the 10 FMGs.

The CNVs and DEGs from the 309 FMGs were detected in the TCGA-KIRC cohort. As a result of exploring the incidence of CNVs, FMGs were found have massive CNV alterations. We have listed the top 10 genes with amplified or deleted CNVs (**Figure 1A**). A total of 34 DEGs were detected in 535 ccRCC samples when compared to 72 normal renal samples. The 10 significantly augmented FMGs were among the DEGs identified in the ccRCC samples, while 24 have been attenuated essentially (**Figures 1B, C**). The OS-related FMGs were screened in TCGA-KIRC and E-MTAB-1980 datasets (**Figures 1D, E**). In total, 160 and 67 significant OS-related FMGs were retrieved respectively. Further analysis of 52 overlapping OS-related FMGs was conducted by combining the results of the two cohorts (**Figure 1F**). Partial likelihood deviation analysis was performed on the results of LASSO regression (**Figures 1G, H**). We calculated the coefficient for the prediction of the prognosis of ccRCC by the OS-related FMGs (**Figure 1I**).

### Effect of expression levels of each of the 10 FMGs in the signature on prognosis of RCC

**Figure 2:**
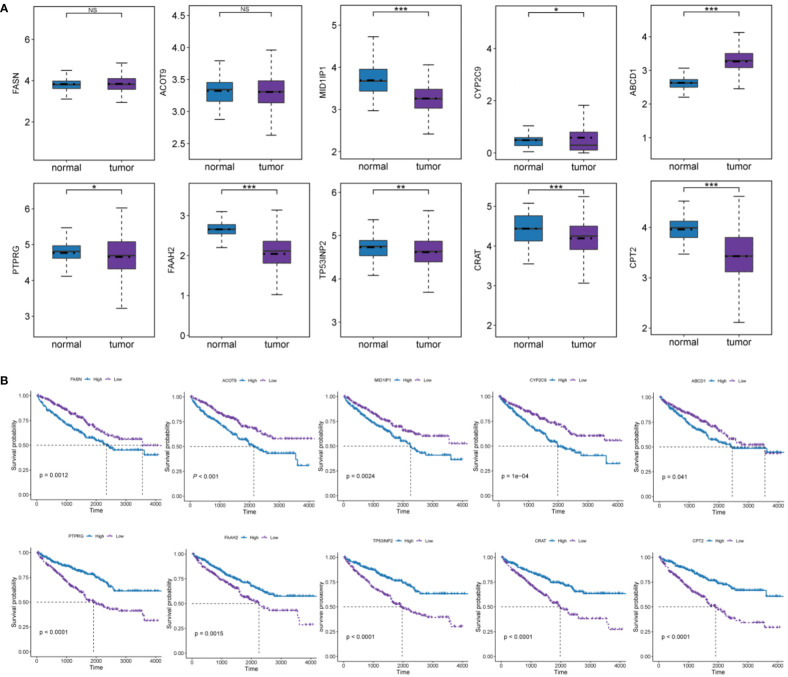
Analyses of the effect of expression levels of each of the 10 FMGs in the signature on prognosis. **(A)** Expression level of the 10 prognostic FMGs in tumor and normal samples. **(B)** K-M overall survival curves of ccRCC patients according to relative expression of the 10 FMGs. *P< 0.05; **P< 0.01; ***P< 0.001; ns means no significance.

A prognostic gene signature was constructed by identifying 10 pivotal OS-related FMGs, namely, FASN, ACOT9, MID1IP1, CYP2C9, ABCD1, CPT2, CRAT, TP53INP2, FAAH2, and PTPRG. The expression level and prognostic potential of the 10 selected genes were evaluated individually. Boxplots were used to depict the expression level of the 10 prognostic FMGs in tumors and normal tissues (**Figure 2A**), and K-M curves were drawn for analysis of OS (**Figure 2B**). As shown in the figures, a significant decrease was observed in the expression of MID1IP1, CYP2C9, CPT2, CRAT, TP53INP2, FAAH2, and PTPRG, while a moderate increase in the expression of ABCD1 was observed in the ccRCC samples. As noted in the separate K-M analyses of OS, high expression of FASN, ACOT9, MID1IP1, CYP2C9, and ABCD1 and low expression of CPT2, CRAT, TP53INP2, FAAH2, and PTPRG were associated with more impaired OS.

### Evaluation and validation of the 10-FMG signature

Based on the expression level of the 10 FMGs, the FMI was calculated using the following formula. FMI = Sum of the expression of each gene × coefficients = FASN × 0.204117 + ACOT9 × 0.151747 + MID1IP1 × 0.149099 + CYP2C9 × 0.147525 + ABCD1 × 0.106468 − CPT2 × 0.20157 − CRAT × 0.222481 − TP53INP2 × 0.240641 − FAAH2 × 0.278899 − PTPRG × 0.314233.

**Figure 3:**
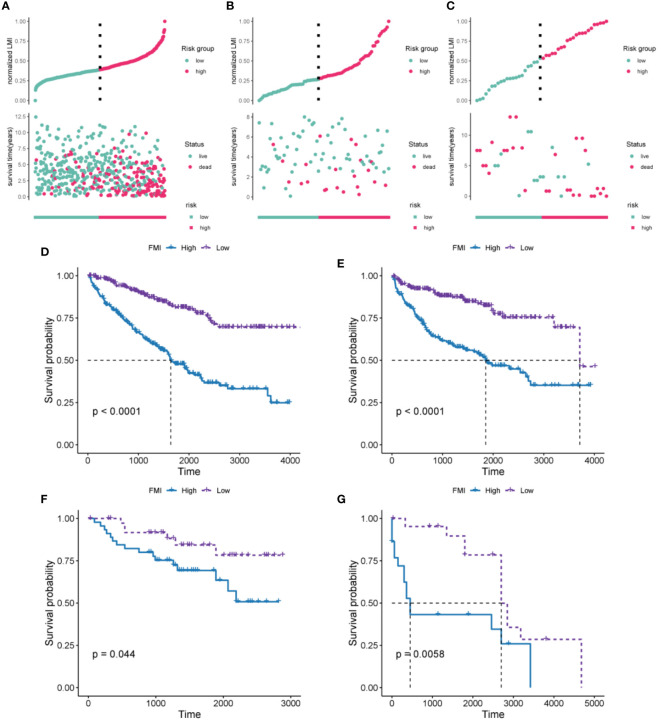
Evaluation and validation of the 10-FMG signature. Distribution plots of the patients’ normalized FMI and OS status TCGA-KIRC **(A)**, E-MTAB-1980 **(B)**, and GSE22541 cohorts **(C)**. K-M analyses of OS **(D)** and DFS **(E)** in the TCGA-KIRC cohorts. K-M analyses of OS **(F)** and DFS **(G)** in the E-MTAB-1980 and GSE22541 cohorts respectively.

According to their median FMI values, ccRCC patients could be classified as low-risk or high-risk group. Further, FMI was normalized for easy visual representation of the data. According to the data for the TCGA-KIRC cohort, patients in the high-risk group were more likely to die than those in the low-risk group (**Figure 3A**). The prognostic significance of FMI was confirmed in two additional cohorts (**Figures 3B, C**). K-M analyses revealed that the high-risk group had significantly worse OS and disease-free survival (DFS) than the low-risk group in TCGA-ccRCC cohort (**Figures 3D, E**). The two additional cohorts showed that OS deteriorated more among those at high risk than those at low risk, consistent with the TCGA-ccRCC cohort (**Figures 3F, G**).

### Correlation between FMI and clinical features of ccRCC

**Figure 4:**
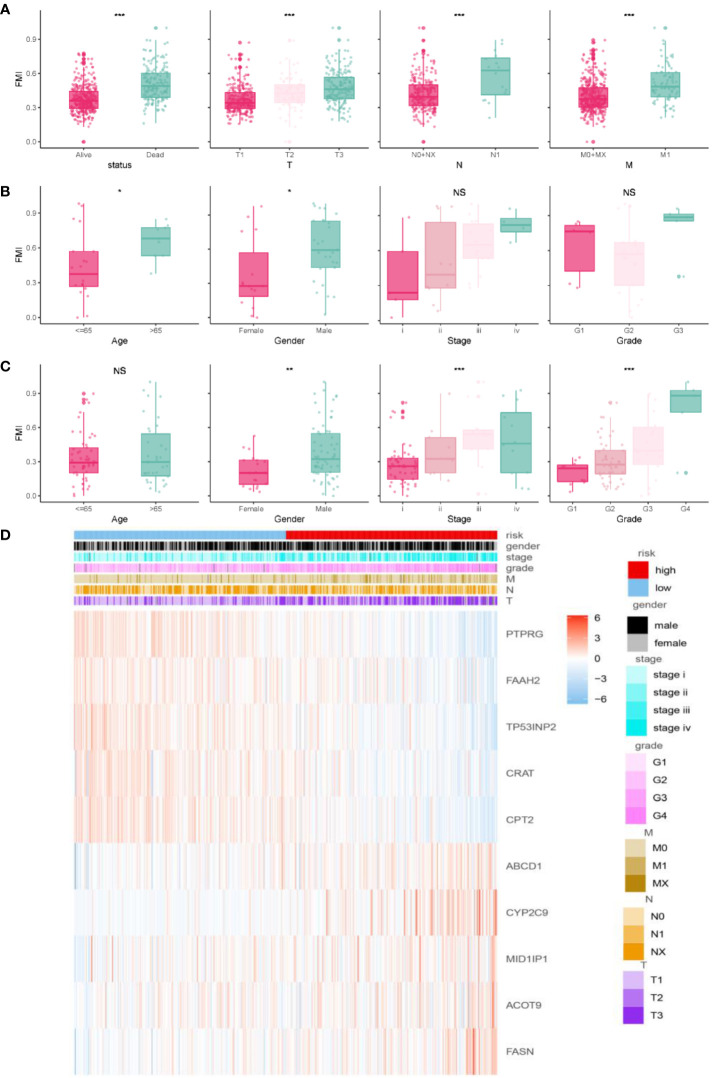
Correlation analysis of clinical features and FMI. Association between adjusted FMI and different clinical parameters in the TGCA-KIRC. **(A)**, E-MTAB-1980 **(B)**, and GSE22541 **(C)** cohorts. Heatmaps of the correlations between FMI and clinical parameters in the TGCA-ccRCC cohort **(D)**. *P < 0.05; **P < 0.01; ***P < 0.001; ns means no significance.

The clinical parameters survival status and clinicopathologic T/N/M were correlated with FMI to varying degrees (**Figure 4A**, *P*< 0.05 for all). That is, higher FMI was associated with greater severity of these clinical characteristics. The E-MTAB-1980 cohort also showed conspicuous differences in various clinical parameters, including tumor stage and grade (**Figure 4B**). In addition, FMI was found to be associated with gender and age: specifically, male patients and patients older than 65 years had higher FMI than female patients and patients younger than 65 years in the E-MTAB-1980 and GSE22541 cohorts (except for age in the GSE22541 dataset) (**Figures 4B, C**). **Figure 4D** presents a heatmap of the overall distribution of the 10 FMGs with clinical parameters in the TCGA-KIRC cohort.

### Establishment and assessment of an FMG-based clinicopathologic nomogram

**Figure 5:**
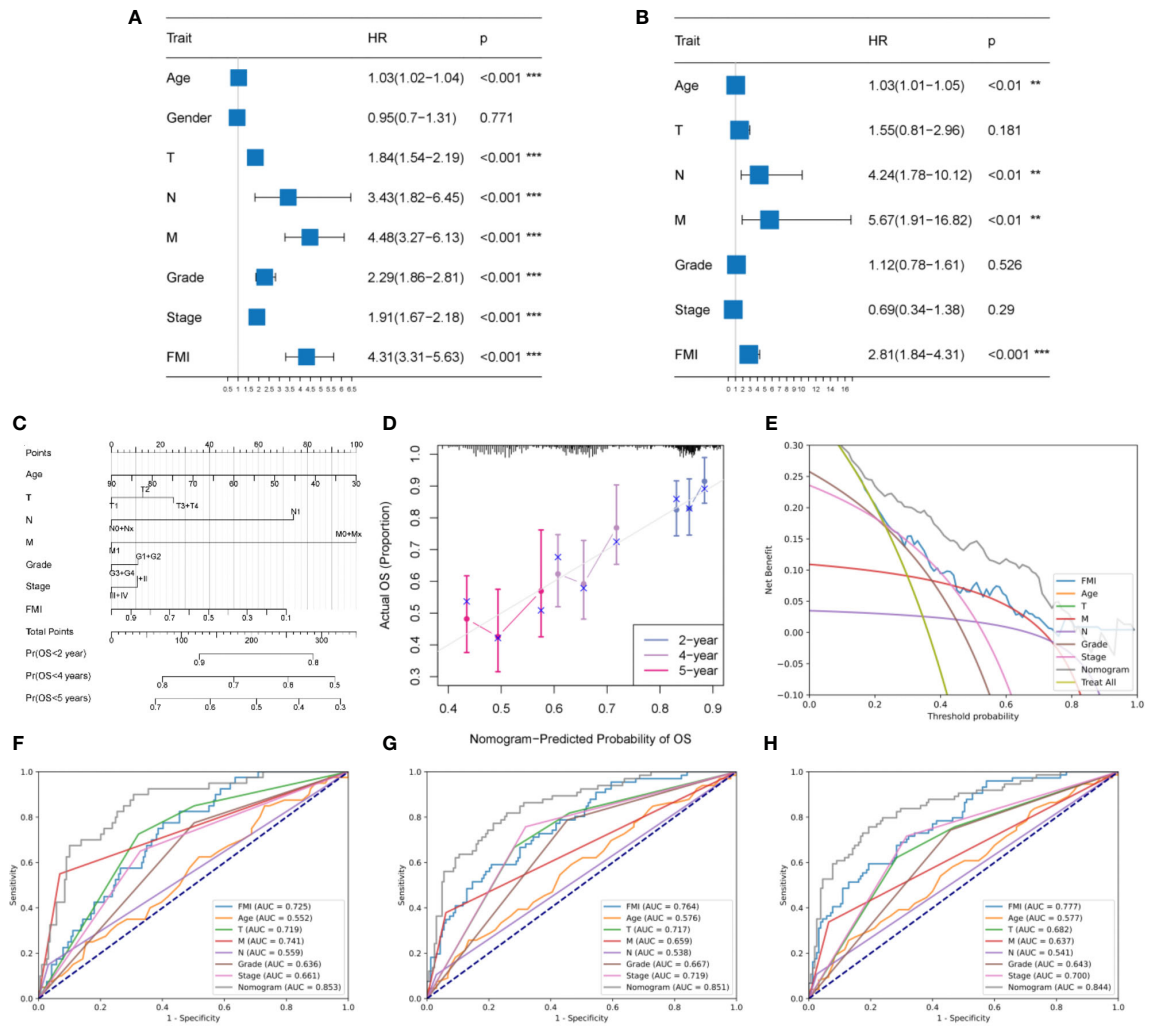
Development and evaluation of a clinicopathologic nomogram based on the identified FMGs. **(A, B)** Univariate and multivariate Cox regression analyses. **(C)** Development of a prognostic nomogram based on age, T stage, N stage, M stage, tumor grade, AJCC stage, and FMI. **(D)** Calibration curve showing the predicted OS versus actual OS. **(E)** DCA of the clinical usefulness of the constructed nomogram. **(F, G, H)** Receiver operating characteristic (ROC) analysis of the nomogram for predicting 2-, 4-, and 5-year OS in the TCGA-KIRC cohorts. **P < 0.01; ***P < 0.001.

According to univariate Cox analysis, age, T/N/M stage, tumor grade, AJCC stage, and FMI showed a remarkable association with OS (**Figure 5A**, *P*< 0.001 for all). Multivariate Cox analysis of these variables showed that only age, N, M, and FMI were independent predictors (**Figure 5B**, *P*< 0.01 for all).

According to the above results, an individual OS prediction nomogram was developed using FMI and the six clinical features that were associated with prognosis according to univariate Cox regression analysis (**Figure 5C**). In the calibration plot, the nomogram was similar to an ideal curve in terms of predictive value, and this was indicative of perfect stability (**Figure 5D**). According to the results of DCA, the nomogram had a better predictive effect than any individual clinical feature (**Figure 5E**). Additionally, the area under the ROC curve values for the nomogram for 2-year, 4-year, and 5-year survival were 0.853, 0.851, and 0.844, respectively, and it had better efficiency than each of the other clinical factors in predicting OS (**Figures 4F, G, H**). Thus, the predictive nomogram for OS appears to be fairly accurate, and it could be used to assist decision-making in the clinical setting.

### GSEA analysis based on FMI grouping

**Figure 6:**
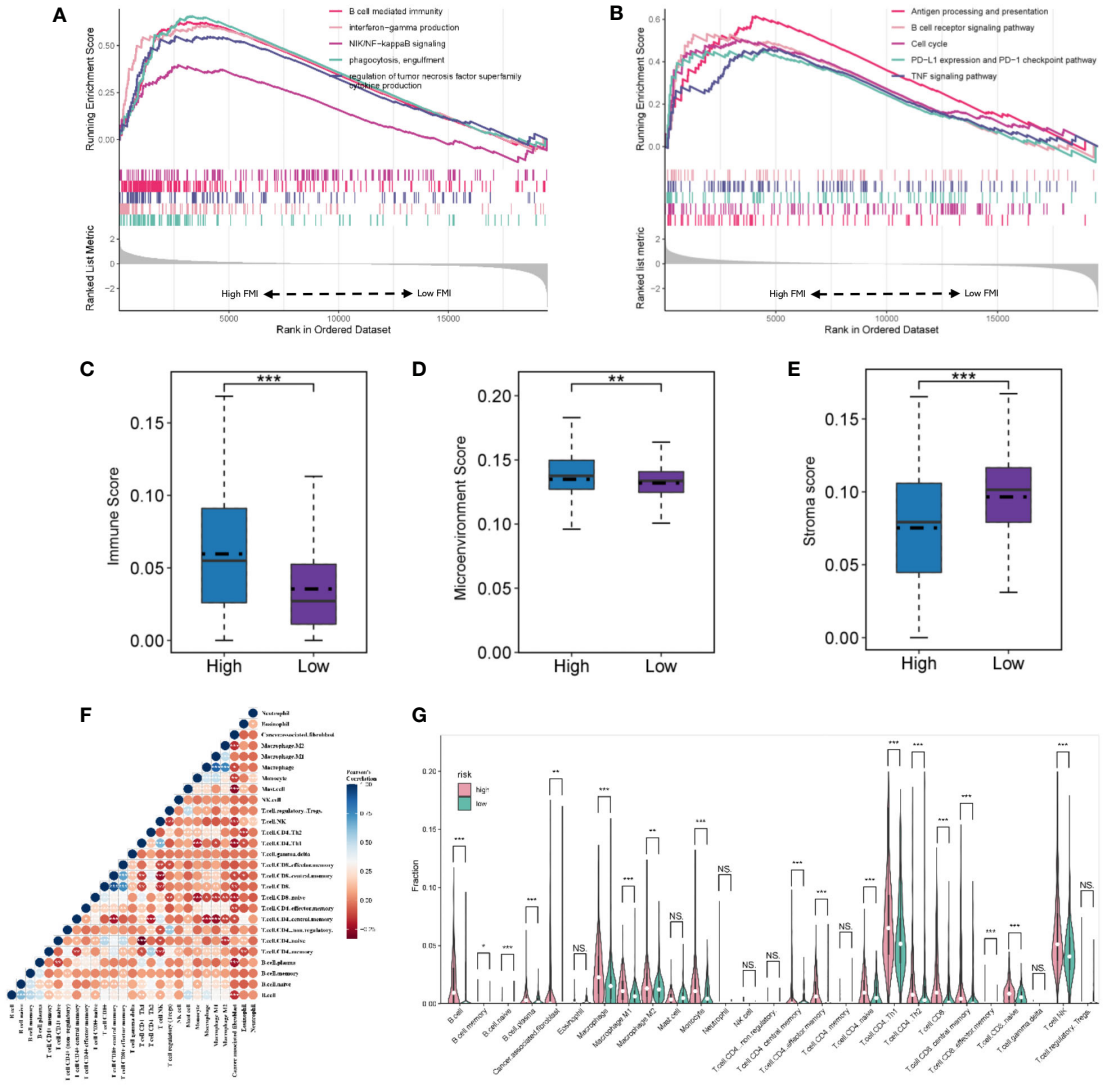
Gene set enrichment analysis and landscape of the immune microenvironment in the TGCA-KIRC. GO **(A)** and KEGG pathway **(B)** analyses of the high- and low-FMI groups. **(C, D, E)** Evaluation of the tumor microenvironment of ccRCC. **(F)** The correlation of infiltrating immune cells. **(G)** Violin diagram of the proportions of different tumor-infiltrating cells in the high- and low-FMI group. *P < 0.05; **P < 0.01; ***P < 0.001. ns means no significance.

The GSEA analysis results from the GO database, demonstrated in **Figures 6A and B**, indicate that B-cell-mediated immunity, interferon−gamma production, NIK/NF−kappaB signaling, phagocytosis, engulfment, and regulation of tumor necrosis factor superfamily cytokine production were considerably enriched in the group with high FMI (**Figure 6A**). In addition, the results from the KEGG database showed that antigen processing and presentation, the B cell receptor signaling pathway, the cell cycle, PD−L1 expression and PD−1 checkpoint pathway, and the TNF signaling pathway were enriched in the high-FMI group (**Figure 6B**).

### Immune microenvironment of ccRCC

In TCGA cohort, the immune score and tumor microenvironment score were higher in the high-FMI group, whereas the stroma score was markedly lower (**Figures 6C, D, E**, *P*< 0.05 for all). The tumor microenvironment analysis results demonstrated that the number of B cells, plasma B cells, M1 and M2 macrophages, monocytes, central and effector memory CD4+ T cells, naive CD4+ T cells, Th1 and Th2 CD4+ T cells, CD8+ T cells, central and effector memory CD8+ T cells, naive CD8+ T cells, and natural killer (NK) T cells was significantly higher in the high-FMI group (**Figures 6F, G**). Additionally, the immune microenvironment analysis results of E-MTAB-1980 and GSE22541 cohorts are shown in **Supplementary Figure 2**. The results revealed that B cells, plasma B cells, M1 macrophages, Th2 CD4+ T cells, and NK T cells were notably strengthened in the high-FMI group in all three cohorts.

**Figure 7:**
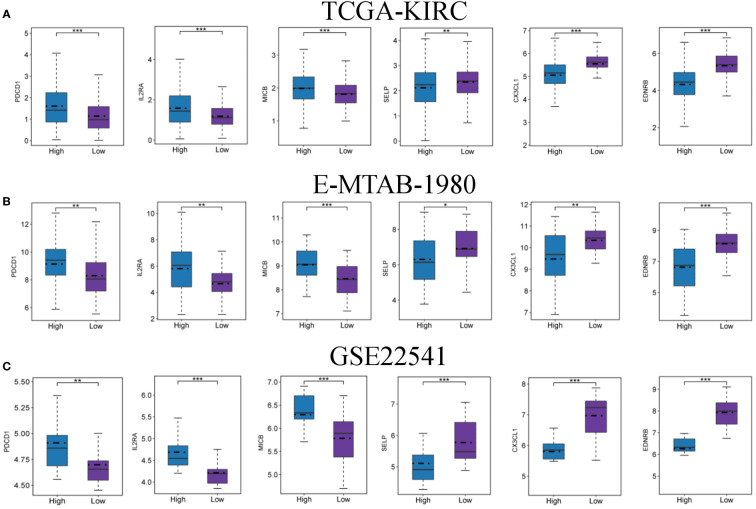
Expression levels of immune checkpoints in the high- and low-FMI group. Expression level of PDCD1, IL2RA, MICB, SELP, CX3CL1 and EDNRB in TCGA-KIRC **(A)**, E-NTAB-1980 **(B)**, and GSE22541 **(C)** cohorts. *P < 0.05; **P < 0.01; ***P < 0.001.

Immunotherapy has shown great promise in cancer treatment, and immune checkpoint blockade is a promising anti-tumor strategy. Accordingly, the expression of six candidate immune checkpoints were assessed. The results revealed that PDCD1, IL2RA and MICB exhibited significant augmentation in the high-FMI group, whereas SELP, CX3CL1 and EDNRB exhibited significant augmentation in the low-FMI group. All results were consistent across all three datasets (**Figures 7A, B, C**). These findings indicate that the efficacy of immunotherapy against different targets for patients with ccRCC may differ according to whether they have high or low FMI.

### Prediction of chemotherapeutic drug sensitivity

**Figure 8:**
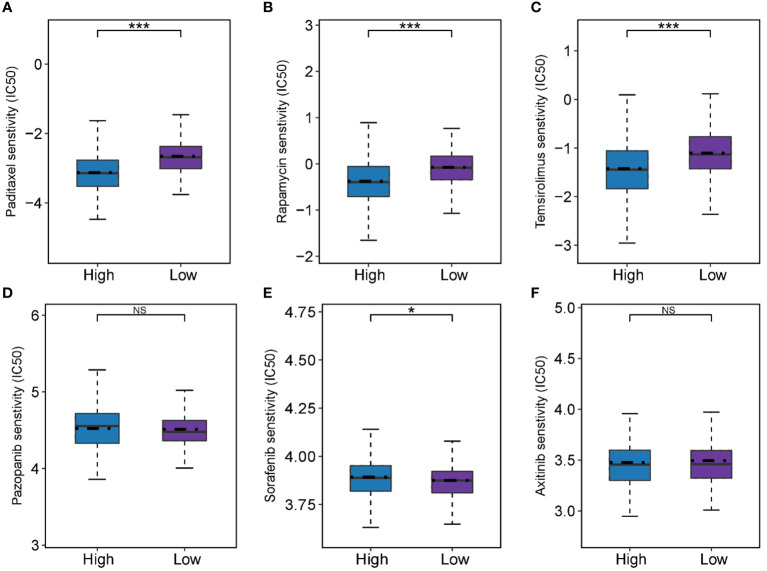
Predictive results of chemotherapeutic responses. **(A-F)** The differences of chemotherapeutic response in the high- and low-FMI group. *P < 0.05; ***P <0.001.

According to the predicted results of the “pRRophetic”, we observed differences in drug sensitivity between different groups (**Figures 8A-F**). The results showed that there were no difference in response for pazopanib and axitinib (*P* > 0.05 for all), and the low-FMI group was more sensitive to sorafenib (*P*< 0.05), while the high-FMI group were more sensitive to paditaxel, rapamycin, and temsirolimus (*P*< 0.05 for all).

### Clinical validation of the expression of genes

**Figure 9:**
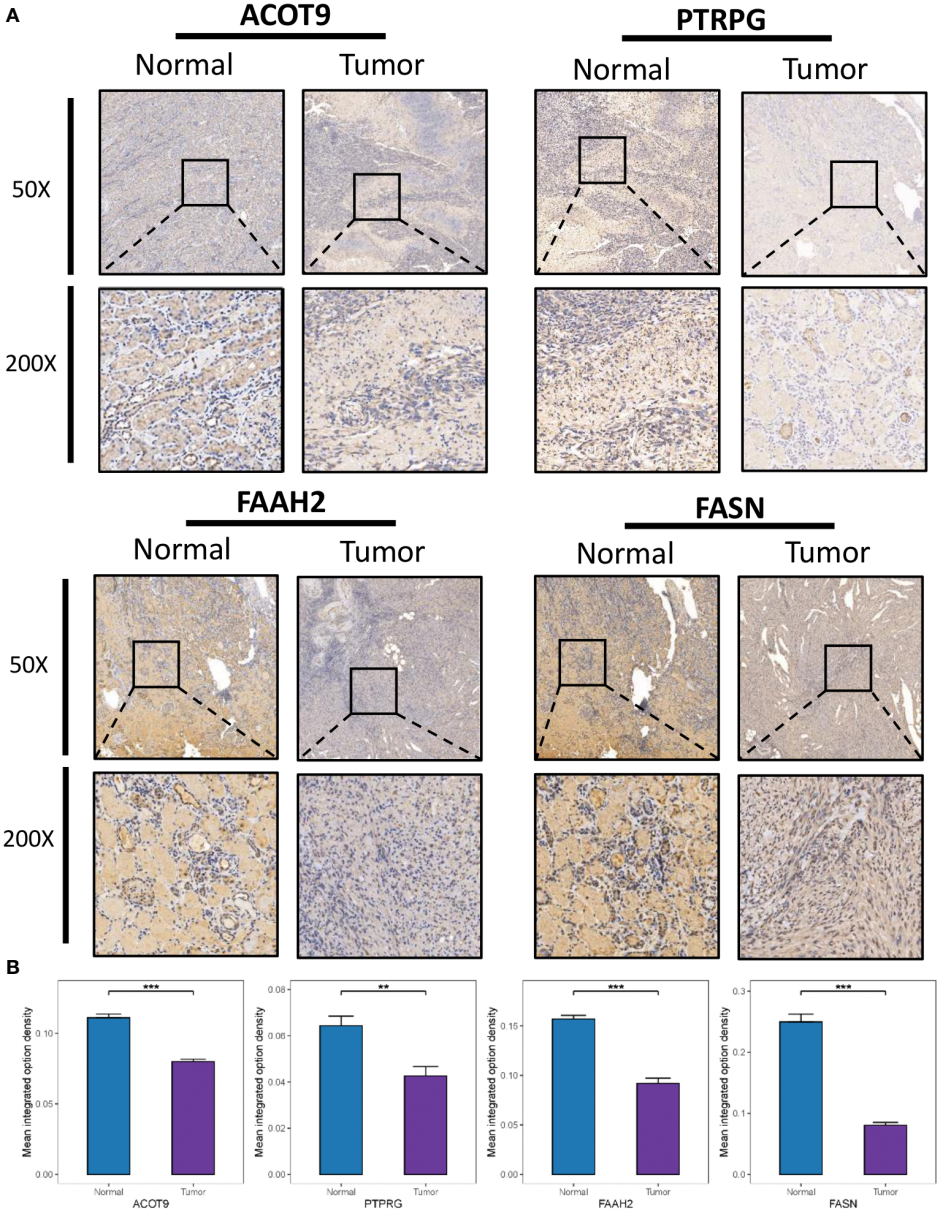
Clinical Validation of the risk model based on IHC. **(A)** Representative IHC images of the four selected gene. **(B)** The quantitative expression levels of each gene. **P < 0.01; ***P < 0.001.

The protein expression of four genes (FASN, ACOT9, FAAH2, and PTPRG) in the identified FMG signature was validated with IHC in 10 ccRCC samples and 10 paired normal samples. The results showed that all the four genes expressed in higher amounts in normal samples than in tumor samples (**Figures 9A, B**). In particular, to our knowledge we evaluated the immunohistochemical expression of FAAH2 in ccRCC for the first time. The protein expression of other 4 genes (ABCD1, CPT2, CRAT and MID1IP1) in the identified FMG signature could be assessed using the Human Protein Atlas (http://www.proteinatlas.org/) database, and we summarized the representative images of these genes in **Supplementary Figure 3**.

### Pain dissection of the FMGs signature

Considering that the majority of cancer patients experience pain during cancer progression or treatment, we further dissected the association of FMGs signature with cancer pain. As shown in **Table 1**, we first provided literature evidence for 10 signatures associated with fatty acids, and further we summarized the literature-reported evidence for pain-related genes, including gene FASN, CYP2C9, ABCD1, CPT2, and FAAH2.

**Table 1:** The summary of the pain dissection of the FMGs signature.

Gene	Association with fatty acid	Association with pain
FASN	FASN is a key enzyme regulating the *de novo* synthesis of fatty acids, which can catalyze acetyl-CoA and malonyl-CoA to produce palmitate. (PMID: 26519059)	Palmitate can activate NF-κB transcription factors and regulate the expression of NMDA receptor subunits. FASN can be used as a therapeutic target to reduce neuropathic pain. (PMID: 25855977)
ACOT9	ACOT9 regulates fatty acid synthesis by catalyzing the hydrolysis of fatty acyl-coenzyme A to form free fatty acid (FFA) and coenzyme A (CoA). (PMID: 36004563)	NA
MID1IP1	The change of MID1IP1 expression can affect the expression of fatty acid synthase (FASN) and induce phosphorylation of Acetyl-CoA carboxylase (ACC), thereby affecting the biosynthesis of fatty acids and triglycerides. (PMID: 34153683, 35916211)	NA
CYP2C9	CYP2C9 is a cytochrome P450 enzyme that has cyclooxygenase activity and catalyzes the oxidation of polyunsaturated fatty acid arachidonic acid to eicosatrienoic acids. (PMID: 30012669)	CYP2C9 can predict the analgesic effect of tramadol and ketorolac. (PMID: 34246203)
ABCD1	ABCD1 gene encodes peroxisome transport protein, which is involved in transporting saturated very long chain fatty acids to peroxidase for β-oxidation. (PMID: 32017990)	Absence of ABCD1 will lead to mechanical allodynia mediated by mechanosensitive ion channels and dysfunction of satellite glial cells. (PMID: 35681537)
CPT2	Fatty acid oxidation (FAO) is a process in which carnitine palmitoyltransferase 1 and 2 (CPT1 and CPT2) transport long-chain fatty acids to the mitochondrial matrix, and then oxidize them to acetyl-CoA, NADH and FADH2 and generate energy. (PMID: 33027638)	CPT2 deficiency may lead to metabolic disorder in the body, causing patients to have diffuse muscle pain symptoms. (PMID: 27034144)
CRAT	Carnitine acetyltransferase (CRAT) is the basic enzyme in carnitine metabolism, which regulates the metabolic flexibility of muscle and increases exercise ability. Carnitine can promote fatty acids to enter mitochondria for oxidative decomposition during fat metabolism, which is helpful to promote the balance of fat metabolism. (PMID: 29444428)	NA
TP53INP2	TP53INP2 mediates peroxisome proliferator-activated receptor gamma (PPARG) regulates macroautophagic/autophagic-dependent mechanism that induce brown fat differentiation and thermogenesis. (PMID: 35947488)	NA
FAAH2	Fatty acid amide hydrolase (FAAH1 and FAAH2) can inactivate endogenous cannabinoid, and monoacylglycerol lipase can hydrolyze to 2-arachidonic glycerol. (PMID: 30070030)	Fatty acid amide hydrolase (FAAH) plays an important role in the hydrolysis and inactivation of endogenous arachidonic ethanolamide (AEA). AEA can protect neurons from inflammatory injury by activating cannabinoid receptors (CB1R and CB2R) and transient receptor TRPV1. FAAH inhibitors may become a safe and reliable new analgesic. (PMID: 34364309, 29017758)
PTPRG	PTPRG is a negative regulator of insulin signal transduction, and insulin can promote the synthesis and storage of fat and reduce free fatty acids in blood. (PMID: 29180649)	NA

NA, missing references.

## Discussion

There is considerable evidence that fatty acid metabolism is severely disrupted in ccRCC; further, the dysregulation of various lipid metabolism pathways that drive lipid deposition is closely related to ccRCC (22). For example, it has been appreciated that elevated lipid storage levels can maintain cell membrane fluidity, thereby enhancing metastatic capacity (23). Timely intervention with therapeutic approaches, such as tyrosine kinase inhibition with sunitinib, pazopanib, and nivolumab, has been found to significantly improve survival in patients with advanced RCC (24). However, the complexity of the tumor microenvironment in ccRCC and the high heterogeneity of individual gene regulation are associated with inadequate treatment response and drug resistance. Given the close association between ccRCC and fatty acid metabolism, a systematic analysis of the role of FMGs in RCC could be helpful for understanding the mechanism of disease progression and for treatment decision-making.

In this study, we first identified FMGs and later confirmed the significant role of FMGs in RCC based on the identification of DEGs with CNV alterations. Based on data from the TCGA-KIRC and E-MTAB-1980 cohorts, univariate Cox analysis along with LASSO Cox regression analysis were used to identify a novel robust prognostic signature of FMGs. Subsequently, the signature was used to classify RCC patients into low- and high-FMI groups and was validated in the three cohorts. Further, each ccRCC patient was further stratified by constructing a risk score model, and the groups showed significant differences in survival and various clinicopathological parameters. In addition, ROC analysis demonstrated the superior performance of our model and indicated that it might be useful for formulating follow-up treatments. We further used xCell to construct the immunogenomic landscape of RCC and explore differences in the distribution of immune cells. Altogether, the results above revealed the prognostic signature of our FMGs has a great promise in ccRCC.

The signature we constructed contains 10 fatty acid metabolism genes, some of which have previously been reported to be associated with multiple cancers. FASN encodes fatty acid synthase, which primarily regulates the deposition of animal liposomes by synthesizing long-chain fatty acids from acetyl-coenzyme A (CoA) and malonyl-CoA. All esterified fatty acids in most tumor cells are synthesized *de novo*. FASN is dysregulated in a variety of cancers, including kidney, liver, lung, and colorectal cancer, and this dysregulation is thought to be associated with the aggressiveness and poor prognosis of cancers (25, 26). The ACOT9 gene encodes acyl-CoA thioesterase 9, which is a well-known key regulator of cellular utilization and regulates intracellular acyl-CoA/fatty acid levels. A recent study found that ACOT9 promoted tumor metastasis and growth by reprogramming lipid metabolism pathways in hepatocellular carcinoma (27). Interestingly, we found that the FASN and ACOT9 genes were significantly downregulated in RCC patients. In the future, we will further study its potential mechanisms in ccRCC. Protein tyrosine phosphatase receptor gamma (PTPRG) is a well-known tumor suppressor in various neoplasms (28). For example, Shu et al. found that PTPRG may play an inhibitory role in breast tumorigenesis by upregulating the p21(cip) and p27(kip) proteins through the ERK1/2 pathway (29). In line with this finding, PTRPG expression was significantly reduced in ccRCC according to the IHC results of this study. In addition, the results of this study revealed that low expression of PTRPG could predict poor prognosis. According to recent reports, other genes, such as MID1IP1 (30), ABCD1 (31), CPT2 (32), and TP53INP2 (33), are closely associated with the progression of ccRCC. However, our study is the first to demonstrate that FAAH2 is inhibited in ccRCC and is an indicator of poor prognosis. In general, the above results confirm the reliability of our signature to a certain extent, but the specific influencing mechanism and prognostic value in clinical practice need to be further studied.

In order to further investigate the role of the signature genes, GSEA analyses were conducted in two FMI groups. Noticeable NIK/NF-κB signaling enrichment was observed in the high-FMI patients. Growing body of research suggests that dysregulation of NF-κB signaling pathway activity can lead to inflammatory diseases as well as cancer and NF-κB has long been proposed as a potential therapeutic target (34). Meteoglu et al. reported that NF-κB was associated with markers of angiogenesis and apoptosis in ccRCC, including VEGF, EGFR, and p53 (35). In addition, it has also been reported that activation of the NF-κB pathway is associated with ccRCC cell migration and invasion (36). Further, drugs that target NF-κB have been found to have therapeutic and preventive effects in a variety of cancers (37, 38). The results of our study suggest that patients with high FMI could benefit more from NF-κB-targeted therapy than patients with low FMI. Similarly, it is now widely accepted that immunotherapy is an effective method for treating cancer, and an increasing number of immunotherapy drugs are being evaluated in clinical trials (39). As an indispensable strategy in immunotherapy, immune checkpoint inhibitors have gained attention for their potential to improve the long-term outcomes of cancer patients (40). However, the effectiveness of this treatment varies, as it is only effective in certain subsets of cancer patients (41). Therefore, we compared six immune checkpoint genes to explore potential immune therapeutic targets in different FMI groups. In the high-FMI group, PDCD1, IL2RA and MICB were significantly elevated, whereas in the low-FMI group, SELP, CX3CL1 and EDNRB were significantly elevated. These results indicate that FMI should be considered when making decisions about immune checkpoint inhibitor therapy for ccRCC patients. Brahmer et al. has reported that PD-L1 inhibitors could promote tumor regression and prolong survival in patients with advanced cancers including ccRCC (42). Accordingly, ccRCC patients with higher FMI might be more likely to benefit from anti-PD-L1 therapy, since they have higher expression levels of PDCD1.

Notably, the majority of cancer patients experience pain during cancer treatment and after curative treatment (55% and 40%, respectively) (43). For cancer survivors, the long-term sequelae of pain after cancer treatment should not be ignored, as cumulative reports have found that opioid abuse is associated with increased mortality (44). Therefore, there is an urgent need to explore other effective pain management options. Basically, cancer cells are abnormal cell growth and proliferation, and fatty acid metabolism changes significantly in the rapid proliferation of cancer cells. Accordingly, interventions to prevent fatty acid synthesis, increase fatty acid degradation through oxidation, and decrease fatty acid release from storage are commonly used to manage the abnormal proliferation of lipids and arrest cancer progression (45). Among the 10 fatty acid metabolism genes associated with prognosis that were identified in this study, FASN has been previously reported as a therapeutic target. That is, studies have confirmed that inhibition of FASN reduced triacylglycerol and phospholipid levels and inhibited lymph node metastasis of prostate carcinoma (46). Similarly, down-regulation of CPT2 also inhibited fatty acid β-oxidation in the tumor microenvironment and promoted cancer progression through acylcarnitine accumulation (47). Interestingly, fatty acid metabolism interventions may not only alter cancer cell proliferation but also help reduce pain during the disease. Recent studies have found that specialized pro-resolving lipid mediators (SPMs) can reduce fatty acid levels and effectively relieve chronic pain, and this mechanism of pain regulation is currently believed to be associated with the activation of immune cell receptors in the lipid environment, changes in pro-/anti-inflammatory pathways, and changes in peripheral nociceptor sensitivity (48). For example, SPMs can activate the immune cell receptor N-formyl peptide receptor 2 (ALX/FPR2), induce cell cycle arrest, and prevent phosphorylation of the nuclear factor kappa B (NF-κB) pathway (49). Moreover, altered fatty acid metabolism may also prevent the formation of neutrophil extracellular traps, thus promoting inflammation resolution and exerting an analgesic effect (48). In a nutshell, our results and the aforementioned studies might indicate that interventions targeting fatty acid metabolism-related genes may have a dual effect on improving prognosis and pain that warrants further investigation.

## Conclusions

In summary, we integrated multiple bioinformatic analysis methods to construct a reliable 10-gene prognostic signature of ccRCC based on fatty acid metabolism and established a nomogram that can be used in clinical practice. The signature may also serve as a potential therapeutic target with dual effects on both ccRCC prognosis and cancer pain, but further studies are needed to support the conclusions.

## Data availability statement

The original contributions presented in the study are included in the article/**Supplementary Material**. Further inquiries can be directed to the corresponding author.

## Author contributions

RD, HW and XJ designed the study and wrote original draft. RD and LW analyzed the data and performed the bioinformatics analysis. MD and HY reviewed the conclusions. RD, HW and XJ edited and revised the manuscript. All authors contributed to the article and approved the submitted version.
