# Supplementary material for: Prognosis and pain dissection of novel signatures in kidney renal clear cell carcinoma based on fatty acid metabolism-related genes
Source: Front Oncol. 2022 Dec 9;12:1094657. doi: 10.3389/fonc.2022.1094657 (PMC9780486; doi:10.3389/fonc.2022.1094657)
Supplement: Supplementary file 1 [file DataSheet_1.docx]

***Supplementary Material***


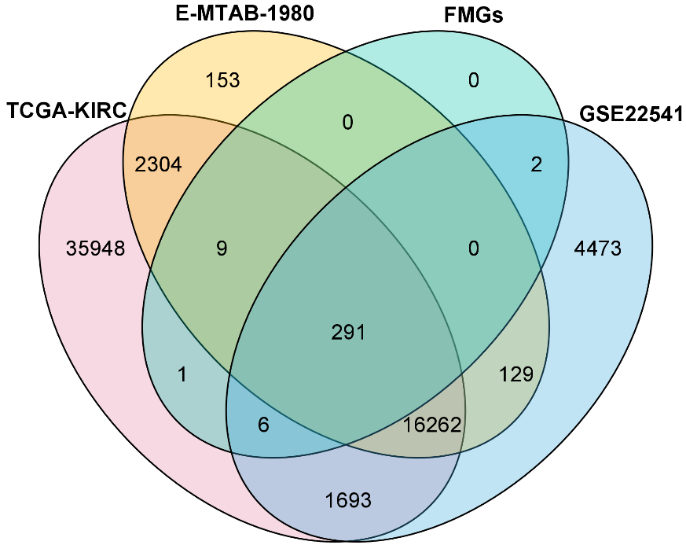


**Supplementary Figure 1.** Venn diagram to identify 291 overlapping FMGs in TCGA-KIRC, E-MTAB-1980 and GSE22541 cohorts.


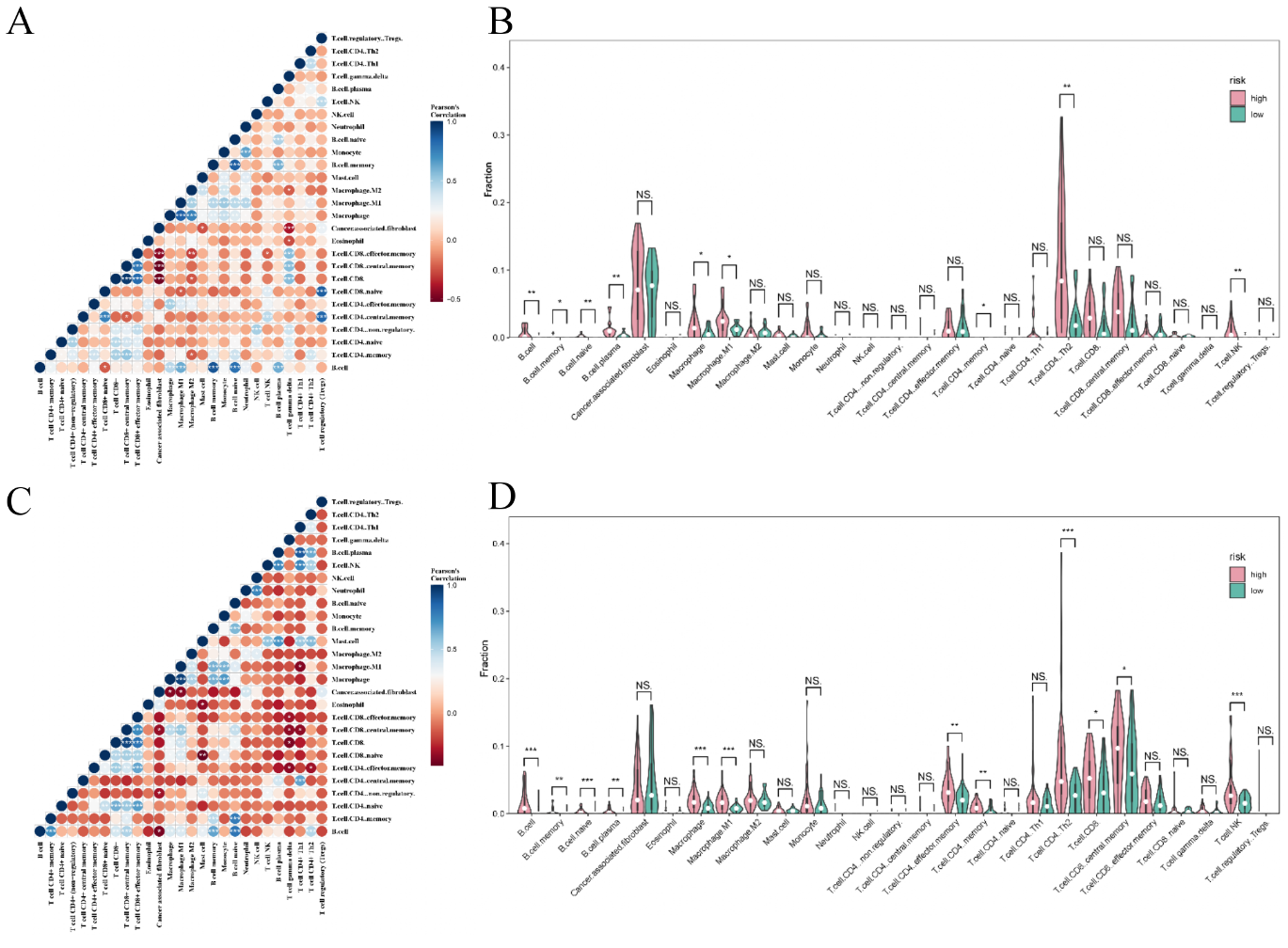


**Supplementary Figure 2.** Landscape of the immune microenvironment. The correlation of infiltrating immune cells in E-MTAB-1980 (A) and GSE22541 cohorts (C). Violin diagram of the proportions of different tumor-infiltrating cells in E-MTAB-1980 (B) and GSE22541 cohorts (D).


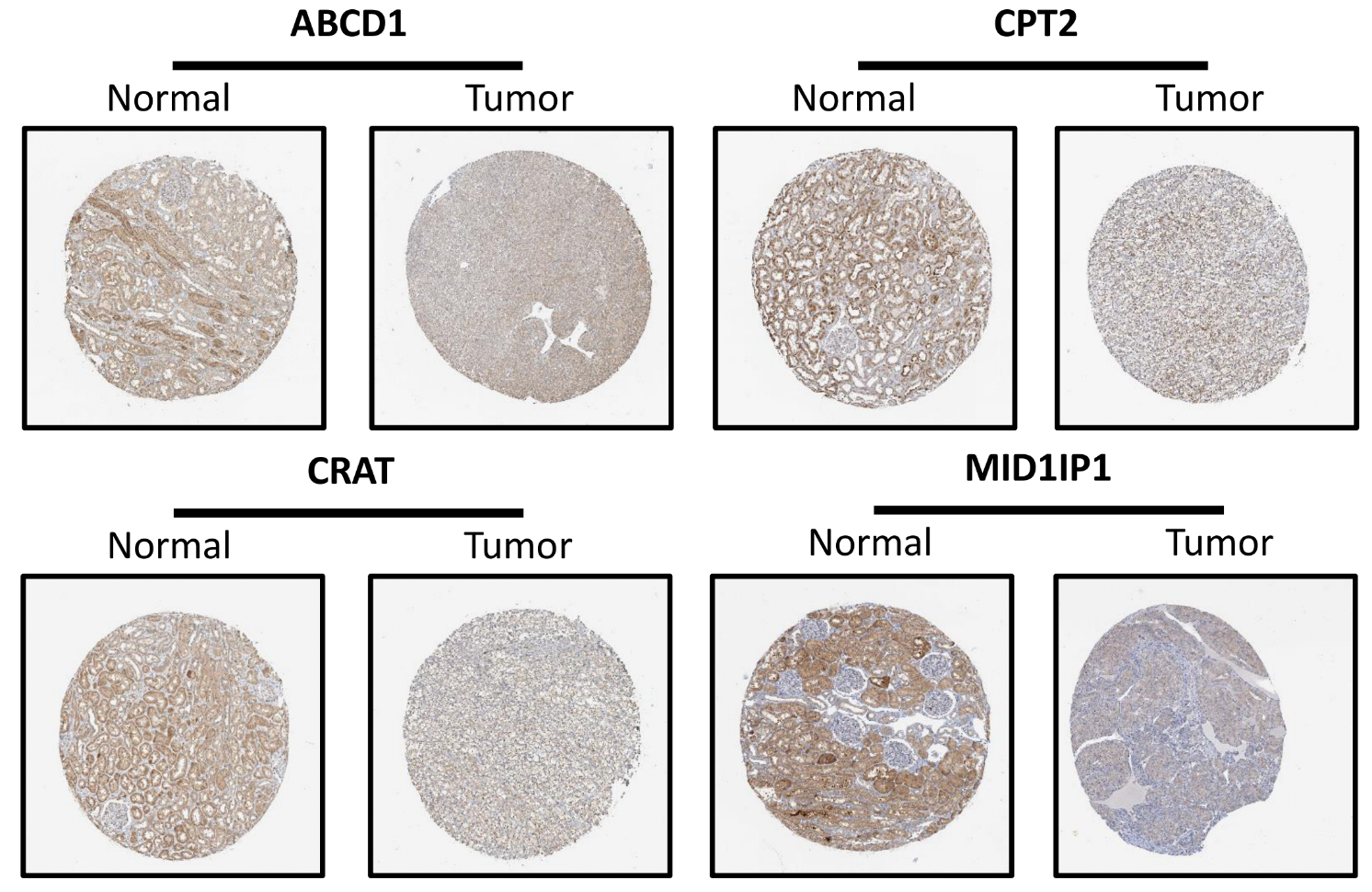


**Supplementary Figure 3.** The representative protein expression of ABCD1, CPT2, CRAT and MID1IP1 gene in ccRCC and normal renal tissue from the Human Protein Atlas database (https://www.proteinatlas.org).

**Supplementary Table 1**. The information of 291 overlapping FMGs.

| Num | Symbol | | | | | | | |
| --- | --- | --- | --- | --- | --- | --- | --- | --- |
| 1 | FAAH | MIF | CYP4F3 | PTGR1 | AADAT | ALDH3A2 | ACBD4 | ABCD1 |
| 2 | ACSM3 | CPT1C | CYP4F8 | PTGR2 | MDH2 | ALDH3A1 | IDH1 | HIBCH |
| 3 | ECI1 | CPT1B | ACADSB | HADHB | PTGES2 | ACLY | UROS | YWHAH |
| 4 | ECI2 | CYP2U1 | PRDX6 | HADHA | PTGES3 | GPD2 | EPHX2 | ADSL |
| 5 | AQP7 | PSME1 | MCAT | UGDH | SCD5 | GPD1 | EPHX1 | HSDL2 |
| 6 | UBE2L6 | SUCLG2 | ALOX15B | ACSBG1 | AKR1C3 | MAPKAPK2 | DHCR24 | HSP90AA1 |
| 7 | ENO2 | ACOT1 | CYP2C9 | PPARA | FMO1 | ERP29 | CYP4F11 | GPX2 |
| 8 | ENO3 | SUCLG1 | CYP2C8 | DLD | G0S2 | GAPDHS | TECRL | PTGIS |
| 9 | IL4I1 | ACOT6 | VNN1 | ACSBG2 | GAD2 | SMS | ACSF3 | GPX1 |
| 10 | NSDHL | ALDH7A1 | FASN | MGLL | CYP8B1 | DPEP2 | ACSF2 | HMGCS1 |
| 11 | LGALS1 | ACOT4 | CYP1A2 | PPARD | FABP1 | DPEP1 | GRHPR | GPX4 |
| 12 | TDO2 | PTGES | CYP1A1 | FH | FABP2 | XIST | AMACR | CYP4A11 |
| 13 | SCP2 | GCDH | PPT1 | ECHS1 | NUDT7 | SLC27A3 | OSTC | BCKDHB |
| 14 | CYP1B1 | SLC22A5 | PPT2 | ADH1C | APEX1 | SLC27A2 | TBXAS1 | FAAH2 |
| 15 | MLYCD | RETSAT | TECR | ADH1B | NDUFAB1 | IDI1 | SERINC1 | PLA2G4A |
| 16 | PTGDS | MAOA | PRKAG2 | ADH1A | CRAT | ALOX15 | ACO2 | INMT |
| 17 | ACAD11 | CD1D | HCCS | ODC1 | PECR | ALOX12 | REEP6 | SLC25A17 |
| 18 | GLUL | ADH7 | CROT | HSD17B3 | ACAA2 | CYP2C19 | BMPR1B | ACOX2 |
| 19 | ACAD10 | ADH5 | PTPRG | DLST | CPOX | CYP4F22 | SLC25A1 | PCCA |
| 20 | ACOT9 | ADH6 | MCEE | HSD17B4 | FADS2 | ADIPOR2 | ACADVL | ACOX1 |
| 21 | CPT1A | ADH4 | ACADL | ALOX12B | HPGDS | ACACB | CYP4A22 | EHHADH |
| 22 | ACOT8 | LDHA | AUH | HSD17B7 | UROD | ACAT2 | PCTP | ALDH1A1 |
| 23 | GABARAPL1 | HSPH1 | ACADM | PTGS2 | NUDT19 | ACACA | DBI | PCCB |
| 24 | PRKAB2 | NTHL1 | CD36 | HSD17B8 | NBN | ACAT1 | ALAD | ACOX3 |
| 25 | ACOT7 | RDH11 | ACADS | PTS | HADH | THEM5 | ACOXL | BLVRA |
| 26 | MECR | RDH16 | CBR1 | PTGS1 | ACAA1 | THEM4 | ALDH2 | ALDH9A1 |
| 27 | ACSL1 | LTA4H | ELOVL1 | RXRA | FADS1 | HACL1 | CPT2 | PON3 |
| 28 | ECH1 | DECR2 | ELOVL4 | ALOX5 | PHYH | MORC2 | CA2 | PRKAA2 |
| 29 | ACSL6 | HAO2 | ELOVL5 | CBR4 | ACOT11 | GGT5 | CA4 | HPGD |
| 30 | ACSL5 | DECR1 | ELOVL2 | CBR3 | ACOT12 | CYP2J2 | IDH3B | PON2 |
| 31 | ACSL4 | S100A10 | ELOVL3 | AOC3 | SDHC | ACBD6 | ME1 | RAP1GDS1 |
| 32 | ACSL3 | NCAPH2 | ELOVL6 | OLAH | SDHD | ACBD5 | CA6 | PON1 |
| 33 | CEL | CYP4F2 | ELOVL7 | MDH1 | SDHA | PDHA1 | HMGCS2 | ETFDH |
| 34 | PDHB | HSD17B12 | HSD17B11 | LTC4S | BPHL | HSD17B10 | CRYZ | HMGCL |
| 35 | ALDH1B1 | AWAT1 | TP53INP2 | D2HGDH | PCBD1 | SLC25A20 | ACSS1 | ALOXE3 |
| 36 | MMAA | IDH3G | CCDC58 | MID1IP1 | CYP4B1 | GSTZ1 | SUCLA2 | SCD |
| 37 | ALOX5AP | THRSP | METAP1 |  |  |  |  |  |
